# Association Between Nutritional Status and Early Postoperative Infection Risk in Kidney Transplant Patients

**DOI:** 10.3390/nu17111935

**Published:** 2025-06-05

**Authors:** Elena González García, Tamara Arroyo, Mercedes Galván, María José Becerra, Margarita Gallego, Israel Mauro, Yanieli Hernández, Almudena Pérez-Torres, María Ovidia López Oliva, María José Santana, Carlos Jiménez

**Affiliations:** 1 Nephrology Department, La Paz University Hospital, 28046 Madrid, Spain; tamara.arroyo@salud.madrid.org (T.A.); mmercedes.galvan@salud.madrid.org (M.G.); mariajose.becerra@salud.madrid.org (M.J.B.); mgallegoperez@salud.madrid.org (M.G.); israel.mauro@salud.madrid.org (I.M.); yanielihermarydelvalle.hernandez@salud.madrid.org (Y.H.); almudenapereztorres@gmail.com (A.P.-T.); mlopezo@salud.madrid.org (M.O.L.O.); mjose.santana@salud.madrid.org (M.J.S.); cjmartin@salud.madrid.org (C.J.); 2 Hospital La Paz Institute for Health Research (IdiPAZ), La Paz University Hospital, 28046 Madrid, Spain; 3 Nutrition Department, Hospital Universitario Santa Cristina, 28009 Madrid, Spain

**Keywords:** kidney transplantation, malnutrition, albumin, infection, hospitalization stay, GNRI, NRI

## Abstract

Malnutrition is one of the stronger predictors of morbi-mortality in end-stage kidney disease patients. Moreover, malnutrition in hospitalized patients severely affects multiple clinical outcomes, increasing the risk of complications. The Nutritional Risk Index and Geriatric Nutritional Risk Index are indexes used to evaluate the risk of malnutrition in hospitalized adults, which have been validated for dialysis patients and have been reported to be a validated prognostic index of nutrition-related morbidity and mortality. **Objectives**: The aim of this study is to evaluate the prevalence of early postoperative infections and their possible relationship with malnutrition in renal transplantation. **Methods**: We conducted a retrospective observational study, including all patients who received a kidney transplant, a total of 140, between January 2020 and December 2023, at a tertiary-level Spanish hospital. **Results**: The average GNRI was 110.1 ± 11.6, equivalent to adequate nutrition, and only 16.4% of patients were at risk of malnutrition. The mean NRI was 111.4 ± 11.8, equivalent to no risk of malnutrition, and only 17.2% of patients had a moderate-to-severe risk of malnutrition. A total of 30 patients (21.4%) required oral nutritional supplementation at discharge, especially modular protein supplements (86.7%), and 52 patients (37.1%) presented an infection during their stay. The most frequent infections were urinary tract infections (69.8% of the total). Malnutrition calculated by the GNRI or NRI correlated to a longer postoperative hospital stay and a higher rate of infectious complications (*p* < 0.05). **Conclusions**: Malnourished patients have a higher risk of early postoperative complications, including infection, and a longer hospitalization stay. The evaluation of nutritional status for the diagnosis and treatment of malnutrition is strongly recommended in ESKD patients on the waiting list for a kidney transplant.

## 1. Introduction

Patients with end-stage kidney disease (ESKD) often present with a deterioration of their nutritional status [1,2], manifested by the consumption of body proteins and energy reserves. Many contributing factors are related to kidney disease, including insufficient food intake due to dietary restrictions and poor appetite, persistent inflammation, increased resting energy expenditure, multiple endocrine disorders, acidosis, and the dialysis technique itself [3]. Malnutrition prevalence in ESKD patients ranges between 18 and 75%, probably due to an absence of standardized definitions and the variety of existing assessment tools [4]. Nutritional status is one of the major modifiable factors affecting the prognosis and outcomes of CKD patients. The presence of malnutrition is one of the main predictors of morbidity and mortality in ESKD patients [5,6]. As recommended by the K-DOQI guidelines [7], the assessment of nutritional status in patients undergoing dialysis should be incorporated into routine clinical evaluation and addressed, when necessary, as it has been shown to improve survival rates, reduce hospital admissions, and decrease comorbidity. However, no single scoring system has been universally endorsed for this patient population. Thus, the selection of an appropriate nutritional screening tool among the various validated methods—such as the Malnutrition Inflammation Score (MIS), the 7-point Subjective Global Assessment (SGA), or the Geriatric Nutritional Risk Index (GNRI)—is at the clinician’s discretion.

The Nutritional Risk Index (NRI), originally described by Buzby et al. [8], was developed to assess the severity of postoperative complications. It is based on two nutritional parameters: serum albumin concentration and weight loss. The NRI is currently employed to evaluate the risk of malnutrition in hospitalized adults [9]. Given the challenges associated with determining usual body weight in hospitalized patients, the Geriatric Nutritional Risk Index (GNRI) modifies the original formula by substituting ideal body weight for usual body weight. Ideal body weight is calculated using the Lorentz formula, which accounts for the patient’s sex and height [10,11]. Both the NRI and GNRI have been validated in dialysis populations. The GNRI is a strong predictor of nutrition-related health outcomes and mortality [12,13]. It offers an easy and objective approach, relying exclusively on body weight, height, and serum albumin levels. Furthermore, it has demonstrated validity in the assessment of malnutrition and the prediction of all-cause mortality and cardiovascular events in patients with chronic hemodialysis, peritoneal dialysis, non-dialysis chronic kidney disease, and heart failure.

Although less commonly utilized than other nutritional indices, the GNRI has shown a statistically significant inverse correlation with the MIS (r = −0.67; *p* < 0.0001) and has demonstrated high interobserver agreement (κ = 0.98) as well as substantial intraobserver reproducibility (κ = 0.82) [14,15].

For patients with ESKD, kidney transplantation is considered the best renal replacement therapy, offering improvements in both quality of life and survival rates. The objective of this study is to analyze the prevalence of early postoperative infections and their possible relationship with malnutrition in renal transplant recipients.

## 2. Materials and Methods

### 2.1. Study Design

We conducted a retrospective observational study, including all end-stage kidney disease patients who received a kidney transplant, a total of 140, between January 2020 and December 2023, at a tertiary-level Spanish hospital.

**Figure 1:**
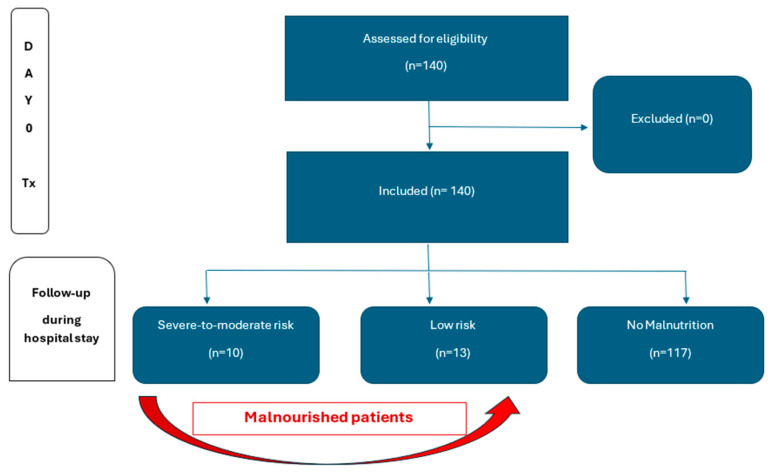
Flowchart study design.

Inclusion criteria were adults aged eighteen and older, who received a living or cadaveric kidney transplant between January 2020 and December 2023 in our center (all the patients who received a kidney transplant during the study period) (Figure 1).

The sample size consisted of all patients who met the inclusion criteria.

### 2.2. Data Collection

All demographic data, including sex, body mass index (BMI), CKD diagnosis, and dialysis history and type, were collected from the medical records. Dialysis duration was defined as the time between the beginning of dialysis and the transplantation date. Patients’ information also included a history of smoking, diabetes mellitus, and systolic blood pressure.

All patients were evaluated at admission (body height and weight) before transplant surgery, and blood samples were collected.

Body height was retrieved from medical records. Body weight and height were measured at admission with patients wearing indoor clothes.

Preoperative blood biochemical indices included serum albumin, prealbumin, protein, creatinine (Cr), C-reactive protein (CPR), phosphorus, and magnesium. These values were obtained from a peripheral blood puncture in the antecubital vein. All tests were performed in the clinical analysis laboratory of the hospital prior to transplantation surgery.

The definitions of NRI and GNRI were as follows:

NRI  =  1.519 × serum albumin (g/L)  +  41.7 × (actual body weight [kg]/ideal body weight [kg]) [8]. Patients were divided into four groups according to their malnutrition status: normal or no nutritional risk (NRI  >  100), mild nutritional risk (NRI 97.5–100), moderate nutritional risk (NRI 83.5–97.5), and severe nutritional risk (NRI  <  83.5).

GNRI  =  1.489 × serum albumin (g/L)  +  41.7 × (actual body weight [kg]/ideal body weight [kg]) [10]. Patients were classified into four groups according to their malnutrition status: no nutritional risk (GNRI  >  98), mild nutritional risk (GNRI 92–98), moderate nutritional risk (GNRI 82–91), and severe nutritional risk (GNRI  <  82).

According to GNRI and NRI definitions, we considered malnourished any patient with a GNRI below 98 or an NRI below 100. Due to the limited available information on the influence of weight changes on nutritional assessment in the early postoperative period after transplantation, we utilized both the NRI and GNRI scores to identify potential differences between the two indices.

### 2.3. Statistical Analysis

The qualitative variables are displayed as absolute frequencies and percentages, while the quantitative variables are presented as means and standard deviations (SD) or as median and interquartile range.

Depending on the data distribution, the qualitative variables were compared between groups using the chi-squared test or Fisher’s exact test. The quantitative variables were compared between groups using the Mann–Whitney U test or Student’s *t*-test, depending on data distribution.

Bivariable and multivariable logistic regression models were used to assess the association with outcomes of interest. Multivariable models included sex, age, type of donor, BMI, albumin and prealbumin levels, immunosuppression, etc. We used a multivariate step logistic regression model, using the conditional forward step method. The results of the model adjustment are presented as odds ratios (ORs), their corresponding 95% CI, and the *p*-values. For differences in hospital stay, groups were tested by multiple linear regression analysis and adjusted by confounding variables.

## 3. Results

### 3.1. General Characteristics of the Population

This study included 140 patients, comprising 53 females (37.9%) and 87 males (62.1%), with a mean age of 53 ± 15 years. The primary renal diagnoses were glomerulonephritis (23.6%, *n* = 33), polycystic kidney disease (17.1%, *n* = 24), diabetic nephropathy (15.7%, *n* = 22), and other/unknown etiologies (43.6%, *n* = 61). During follow-up, two patients died.

Regarding renal replacement therapy (RRT), 65% of the patients received hemodialysis, and 26.4% underwent peritoneal dialysis, while 8.6% had received a transplant prior to RRT initiation. The median time on RRT was 35 months, with an interquartile range between 19 and 48 months.

Table 1 describes the baseline patient characteristics. The median hospital stay was 13 days. When stratified by donor type, the median stay was 8 days for living donors, 14 days for brain-dead donors, and 15 days for controlled circulatory death donors.

No significant sex-based differences were observed in demographic or laboratory characteristics, except for lower mean prealbumin levels in women (25.9 ± 7.0 mg/dL vs. 30.6 ± 9.7 mg/dL, *p* < 0.05).

### 3.2. Prevalence of Malnutrition

Mean preoperative albumin levels were 4.1 ± 0.5 g/dL, with 20 patients (14.3%) presenting hypoalbuminemia (Albumin < 3.4 g/dL). The median prealbumin value was 28.1, with an interquartile range between 21.85 and 33.63 mg/dL. Before the transplant surgery, the median protein value was 7 g/dL, with a range of 6.4 to 7.6 g/dL.

**Figure 2:**
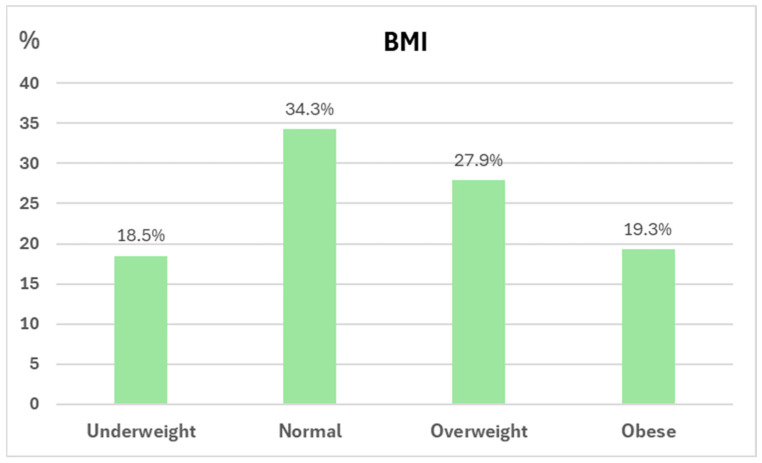

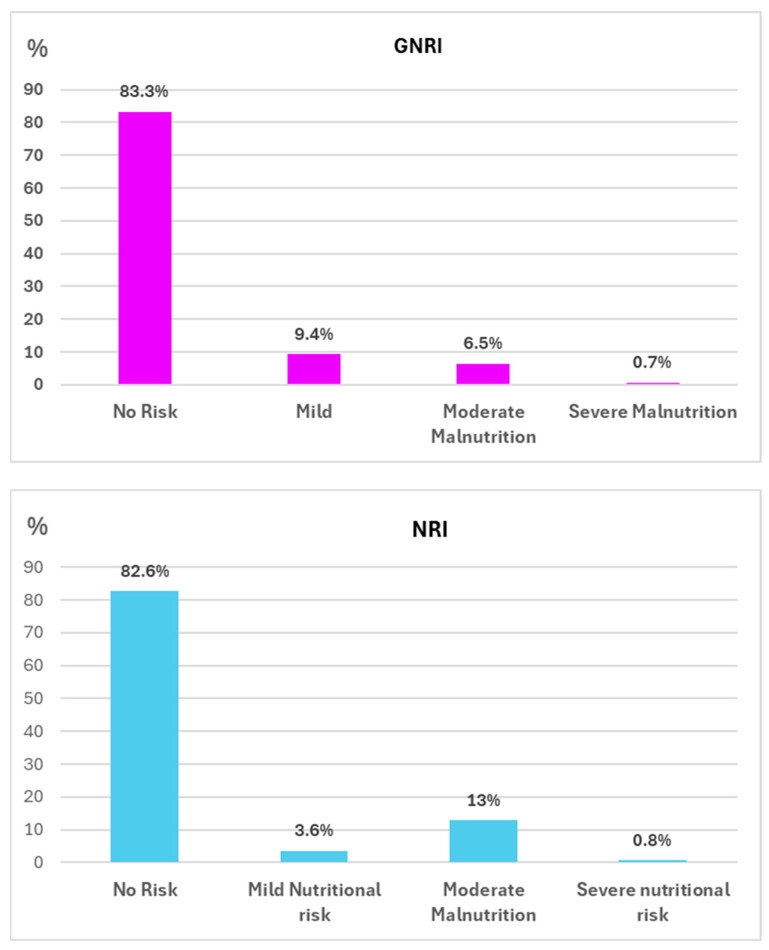
Histogram figures of nutritional status defined by BMI, GNRI, and NRI.

Figure 2 shows the histogram curves of BMI, NRI, and GNRI distribution. The average GNRI was 110.1 ± 11.6, equivalent to adequate nutritional status, and only 13 and 10 patients had a low and severe-to-moderate risk of malnutrition, respectively. The mean NRI was 111.4 ± 11.8, equivalent to no risk of malnutrition, and only 5 and 19 patients had a mild and moderate-to-severe risk of malnutrition, respectively. By NRI and GNRI formulas, 17.4% and 16.7% of the patients had moderate to severe malnutrition, respectively.

A total of 30 patients (21.4%) required oral nutritional supplementation at discharge, especially modular protein supplements (86.7%).

### 3.3. Kidney Transplantation Information

A total of 123 patients (87.9%) received their first kidney transplant, 15 (10.7%) a second one, and 2 patients (1.4%) had a third or more transplants; 21 (15%) were living donor transplants, and 23 patients were hyperimmunized patients with more than 85% of a panel reactive antibody (PRA).

The average cold ischemia time was 12.5 ± 6.9 h for cadaveric transplant, and 65 patients (46.4%) presented delayed graft function (DGF). According to the type of kidney donor, DGF incidence in living donors was 10% (2 out of 20), 60.8% in cadaveric brain death donors (31 out of 51), and 54.2% for controlled cardiac death donors (32 out of 59). The incidence of DGF, defined as the need for dialysis in the first week after transplantation, was 5.3% (1 patient) in living donors, 34.6% in brain death (18), and 49.2% (29) in asystole.

Regarding immunosuppression drugs, induction therapy was based on anti-thymocyte globulins (ATG) in 71.4% of the patients (100) and basiliximab in the rest (living transplant, neoplasia record, etc.), according to our center protocol. Baseline immunosuppression was based on steroids, tacrolimus (target 8–10 ng/mL), and mycophenolate; however, 33 patients (23.6%) received mammalian target of rapamycin (mTOR) inhibitors instead of mycophenolate.

Only 7 patients (5%) presented an acute rejection, and all of them responded to treatment with a bolus of corticosteroids.

### 3.4. Clinical Associations of Malnutrition

Several postoperative complications were evaluated, including in-hospital mortality, and its potential association with nutritional status. During the study, 52 patients (37.1%) developed infections during their hospitalization. The most frequent infections were urinary tract infections (69.8% of cases).

Statistical analysis revealed that malnutrition assessed by the GNRI or NRI was associated with a longer postoperative hospital stay and a higher rate of infectious complications (*p* < 0.05).

Subsequently, we performed a univariate logistic analysis for adverse clinical events. Variables with *p* < 0.05 in univariate analysis were included in multivariate logistic regression. The results showed that malnutrition defined by the GNRI and NRI was an independent risk factor for postoperative infection (Table 2). However, only malnutrition defined by the GNRI, hyperimmunized status, and the length of hospitalization remained statistically significant in the multivariate analysis (Table 3). A hierarchical regression analysis was conducted to examine whether the GNRI provides additional predictive value for postoperative infection risk, beyond established nutritional biomarkers (albumin, prealbumin) and age. The addition of the GNRI did not significantly improve the model’s predictive ability (ΔR^2^ = 0.006, F(1,118) = 0.92, *p* = 0.340). The GNRI coefficient was non-significant (β = 0.09, *p* = 0.340), indicating it failed to provide meaningful incremental predictive value beyond the existing biomarkers.

## 4. Discussion

In this study, we analyzed the prevalence of malnutrition defined by the GNRI and NRI in early postoperative kidney transplantation patients and its relationship with the incidence of infections and hospitalization stay. We observed that 16.4% of patients were malnourished or at risk of malnutrition while on the kidney transplant waiting list. To the best of our knowledge, this is one of the first studies to use the GNRI as a nutritional assessment tool in hospitalized transplant patients. The GNRI is a simple, objective score that correlates strongly with clinical outcomes. We found no significant differences between the NRI and GNRI, suggesting that either index could be used in clinical practice, depending on weight data availability. Although the GNRI did not demonstrate incremental predictive value beyond albumin in our study, it remains valuable in clinical contexts where albumin levels are affected by inflammation or fluid overload.

Although other studies, such as those by Lorden [16] or Neto [17], have analyzed the association between malnutrition and adverse outcomes in kidney transplantation, they primarily focus on the long-term effects of malnutrition in transplant recipients, such as graft loss or reduced quality of life. In contrast, our study shows that nutritional status affects clinical outcomes in the immediate postoperative period, increasing the risk of infection and prolonging hospitalization.

Patients with ESKD have an elevated risk of malnutrition due to multiple etiologic factors, including not only dietary restrictions and poor appetite but also endocrine alterations, persistent inflammation, acidosis, energy losses during dialysis, and an increased resting energy expenditure. In 2008, the International Society of Renal Nutrition and Metabolism (ISRNM) proposed a new nomenclature and diagnostic criteria for malnourished ESKD [18], named protein-energy wasting (PEW). PEW is a condition characterized by reduced body reserves of protein and energy (fat mass). PEW syndrome diagnosis is based on the evaluation of four categories: biochemical criteria; low body weight or weight loss; a decrease in muscle mass; and low protein or energy intake. These four categories could be evaluated by different tools, and at least three out of the four categories must be altered for diagnosis. However, this evaluation is impractical for routine clinical practice. Consequently, malnutrition prevalence in ESKD patients has historically been underestimated due to the lack of standardized definitions, variability in assessment methods and cut-off values, small sample sizes, and differences in socioeconomic conditions across study populations.

A Catalan study [19] reported a malnutrition prevalence of 25.3% in HD and 10.2% in PD, consistent with the results obtained by Antón-Pérez et al. in the Canary Islands [20], who showed that 23% of 468 prevalent HD patients met the criteria for PEW based on ISRNM criteria. Gracia-Iguacel and colleagues, in a Spanish cohort of 122 prevalent hemodialysis patients from Madrid [21], found that 37% of patients at baseline had malnutrition by the PEW criteria. The overall prevalence of malnutrition in our cohort was slightly lower (around 17%), and we found no differences by RRT modality but did observe a significant association with longer dialysis vintage, consistent with the Canarian study findings.

In these cohorts, similar to our study, no significant differences were observed by gender; however, elderly patients were more susceptible to malnutrition development.

Malnutrition in hospitalized patients significantly impacts clinical outcomes. It impairs different mechanisms of the immune system (like innate and cellular immunity), reducing its effectiveness and increasing the risk of infection. Malnutrition prevalence increases with age, care intensity, and comorbidities; however, malnutrition often remains underdiagnosed and undertreated in the hospital. Approximately 20 and 50% of patients are malnourished at admission [22], and about one-third of admission patients with normal nutritional status develop malnutrition during hospitalization [23]. The PREDyCES study [24] was a Spanish nationwide, multicenter, observational study in routine clinical practice, which evaluated the prevalence of hospital malnutrition both at patient admission and discharge. It analyzed the incidence of the complications associated with malnutrition and its relationship with excess hospital stay and healthcare costs. It included 1707 hospitalized patients, and malnutrition was observed in 23.7% of patients, with a longer mean hospital stay with respect to non-malnourished patients. Notably, 9.6% of patients developed malnutrition during hospitalization, while 72% of initially malnourished patients remained so throughout their stay. These findings highlight the need for reliable screening tools to identify at-risk patients for timely nutritional intervention. Similarly, in our cohort, 17% of patients presented malnutrition at admission, and malnourished patients had a longer stay than non-malnourished ones and presented higher rates of complications. In our study, 21.4% of patients needed some kind of oral supplementation at discharge, even with the improvement of appetite and the suppression of dietary restrictions.

Infections remain a frequent complication of renal transplantation. Early post-transplant infections are those that occur in the first 30 days post-surgery and are typically healthcare-associated infections [25]. Most of these infections are post-surgical infections, including surgical site infections, urinary tract infections (UTIs), bacteremias, pneumonias, and Clostridioides difficile colitis. The risk factors for postoperative infections are multifaceted and can be classified into patient-related or procedure-related categories. Patient-related factors include age, obesity, diabetes, malnutrition, steroid use, immunosuppressive state, prolonged hospitalization, procedure site, and coexisting infections at distant sites.

Few studies have analyzed the impact of pre-transplantation malnutrition risk on the clinical outcomes and survival of kidney transplant patients. Ribeiro de Oliveira et al. [26] analyzed in a cohort of 451 patients, the incidence of post-transplant infection depending on their nutritional status and found that the incidence was higher (35.1%) in patients with elevated risk of malnutrition, similar to our findings (37.1%). The most common infections in our cohort were UTIs (almost 70% of all infections). Nutritional status (assessed by the GNRI, NRI, albumin, or prealbumin), age, dialysis vintage, hyperimmunized status, DGF, and hospitalization stay were statistically significantly related to the risk of postoperative infection in the univariate analysis; however, only hospitalization stay, hyperimmunized status and the GNRI remained at a significant value in the multivariate analysis. We did not find any statistically significant relationship between induction or immunosuppressive treatment and infection risk. Hyperimmunized status presented a higher risk of postoperative infection in the univariate analysis, probably due to a more intensive immunosuppressant treatment.

Several limitations should be noted when interpreting these findings. This was a retrospective, observational study based on routine clinical practice, so we lacked additional body composition data (such as bioimpedance) or information about patients’ nutritional status and dietary intake during dialysis. Additionally, albumin levels are influenced not only by hepatic production but also by inflammation and fluid balance. Inflammation reduces albumin synthesis via cytokines, while fluid overload (e.g., post-surgical patients or those with kidney disease) leads to albumin dilution due to plasma expansion. Furthermore, regional dietary patterns may affect malnutrition prevalence. There may be a possible selection bias because more clinically ill patients on dialysis were excluded from the transplant waiting list. Finally, the short follow-up period and absence of long-term complication data for malnourished patients represent important constraints.

## 5. Conclusions

Malnourished patients have a higher risk of early postoperative infectious complications and a longer hospitalization stay. The assessment of nutritional status for the detection and management of malnutrition is strongly recommended in ESKD patients on the waiting list for a kidney transplant.

## Figures and Tables

**Table 1:** Baseline characteristics of participants (*n*-140) according to nutritional status.

	Total Sample (*n* = 140)	No Malnutrition (GNRI > 98) (*n* = 117)	Malnutrition (GNRI < 98) (*n* = 23)
Comorbidities, *n* (%)			
Hypertension	125 (83.9%)	18 (78.3)	105 (91.3)
Diabetes mellitus	33 (23.6%)	6 (26.1)	25 (21.7)
Dyslipidemia	103 (73.6%)	14 (60.9)	87 (75.7)
CKD etiology, *n* (%)			
Glomerular disease	33 (23.6%)	3 (13)	30 (26.1)
Polycystic kidney	24 (17.1%)	5 (21.7)	19 (16.5)
Diabetic nephropathy	22 (15.7%)	3 (13)	17 (14.8)
Interstitial kidney disease	6 (4.3%)	2 (8.7)	4 (3.5)
Unknown	20 (14%)	4 (17.4)	16 (13.9)
Other	26 (18.6%)	5 (21.7)	21 (18.3)
Body mass index, mean (SD), kg/m^2^			
Underweight, *n* (%)	26 (18.6%)	10 (43.5) *	16 (13.9)
Normal	48 (34.3%)	10 (43.5) *	38 (33)
Overweight	39 (27.9%)	3 (13) *	35 (30.4)
Obesity	27 (19.3%)	0 *	26 (22.6)
Barthel index (dependent, score < 90), *n* (%)	13 (18%)	2 (12.6)	13 (18.2)
Renal replacement therapy, *n* (%)			
Hemodialysis	91 (65%)	17 (73.9)	72 (62.6)
Peritoneal Dialysis	37 (26.4%)	6 (26.1)	31 (27)
CKD stage 5	12 (8.6%)	0	12 (10.4)
Time in RRT (months)	35 (307)	37 (307)	34 (97)
GNRI score	110.1 ± 11.6	91.9 ± 5.1 *	113.9 ± 8.8
NRI score	111.4 ± 11.8	92.9 ± 5.2 *	115.2 ± 8.8
Laboratory			
Albumin, g/dL	4.1 ± 0.5	3.36 ± 0.37	4.28 ± 0.43
Prealbumin, mg/dL	28.7 ± 9.0	21.73 ± 6.66	30.1 ± 8.7
CRP, mg/L	2.1 (45.6)	2.8 (24.2)	1.9 (45.6)

Barthel Index for Activities of Daily Living is a tool used to assess the degree of assistance required by an individual. * Statistically significant *p* < 0.05.

**Table 2:** Summary table of significance of clinical events in postoperative infection (univariate analysis).

	OR	OR (95% CI)	*p* Value
Age (per 1-year increase)	1.04	(1.01–1.06)	0.01
Hyperimmunized	3.4	(1.3–8.3)	0.015
Albumin (per 1 g/dL increase)	0.26	(0.13–0.53)	<0.001
Prealbumin (per 1 mg/dL increase)	0.95	(0.91–0.99)	0.025
Dialysis duration (per 1-month increase)	1.04	(1.02–1.06)	<0.001
GNRI (per 1-point increase)	0.97	(0.94–1.00)	0.05
NRI (per 1-point increase)	0.97	(0.94–1.00)	0.049
Hospital stay (per 1-day increase)	1.12	(1.06–1.19)	<0.001
Delayed graft function	3.19	(1.51–6.75)	<0.05

**Table 3:** Summary table of significance of clinical events in postoperative infection (multivariate analysis).

	Β (SE)	Adjusted HR (95% CI)	*p* Value
Hospital stay (days)	0.016 (0.003)	1.02 (1.01–1.02)	<0.001
Hyperimmunized	0.225 (0.105)	1.25 (1.02–1.54)	0.033
GNRI (>98)	−0.265 (0.099)	0.77 (0.63–0.94)	0.009

β = regression coefficient; SE = standard error; HR = Hazard ratio; CI = confidence interval. The multivariate model included all variables listed in the table and was additionally adjusted for sex, age, albumin, prealbumin, hyperimmunized status, dialysis type and duration, GNRI, and delayed graft function. Variables were selected a priori based on clinical relevance and univariate associations (*p* < 0.5). Note. Model shows no multicollinearity (all VIF < 1.2). Coefficients represent changes in log-odds per unit increase. Multicollinearity among predictor variables was assessed using the variance inflation factor (VIF) and tolerance. A VIF value greater than 5 (or tolerance below 0.2) was considered indicative of significant collinearity. If collinearity was detected, remedial measures, such as variable removal or principal component analysis, were considered. Initial collinearity assessment revealed high multicollinearity between GNRI and NRI. To address this, NRI was excluded from the analysis.

## Data Availability

The raw data supporting the conclusions of this article will be made available by the authors on request. Due to Spanish personal and medical data protection laws, we can use or share upon request medical data for scientific purposes but cannot be openly available in internet.
